# Network analysis and experimental pharmacology study explore the protective effects of Isoliquiritigenin on 5-fluorouracil-Induced intestinal mucositis

**DOI:** 10.3389/fphar.2022.1014160

**Published:** 2022-10-06

**Authors:** Yi-fan Liao, Feng-lin Luo, Shan-shan Tang, Jing-wei Huang, Ying Yang, Shuang Wang, Tang-yu Jiang, Qiong Man, Sha Liu, Yi-ying Wu

**Affiliations:** ^1^ Department of Pharmacology, School of Pharmacy, Chengdu Medical College, Chengdu, Sichuan, China; ^2^ Department of Pharmacy, Study on the Structure-Specific Small Molecule Drug in Sichuan Province College Key Laboratory, Chengdu Medical College, Chengdu, Sichuan, China; ^3^ Key Laboratory of Coarse Cereal Processing, Ministry of Agriculture and Rural Affairs, Sichuan Engineering & Technology Research Center of Coarse Cereal Industralization, Chengdu University, Chengdu, Sichuan, China

**Keywords:** 5-fluorouracil, intestinal mucositis, Isoliquiritigenin, inflammatory mediator, network analysis

## Abstract

5-fluorouracil (5-FU) is one of the most widely used chemotherapy drugs for malignant tumors. However, intestinal mucositis caused by 5-FU is a severe dose-limiting toxic effect and even leads to treatment interruption. Isoliquiritigenin (ISL) is one of the main active compounds of licorice, which is a traditional Chinese herbal medicine commonly used in inflammation and gastrointestinal diseases. It is speculated that ISL have protective effects on intestinal mucositis. However, no such studies have been reported. Therefore, to investigate the impact of ISL on 5-Fu-induced intestinal mucositis, a strategy based on network prediction and pharmacological experimental validation was proposed in this study. Firstly, the targets and mechanism of ISL in alleviating 5-Fu-induced gastrointestinal toxicity were predicted by network analysis. And the results were further confirmed by molecular docking. Then, a mouse model of intestinal mucositis was established by intraperitoneal injection of 5-FU (384 μmol/kg) to verify the prediction of network analysis. The network analysis results suggested that PTGS2 (Prostaglandin G/H synthase 2) and NOS2 (Nitric oxide synthase, inducible) might be the critical targets of ISL for reducing the intestinal toxicity of 5-FU. In addition, KEGG and GO enrichment analysis revealed that the HIF-1, TNF, MAPK, IL-17, PI3K-Akt, Ras, NF-kappa B signaling pathway, and biological processes of the inflammatory response, apoptosis regulation, NO production and NF-kappa B transcription factor activity might be involved in the mechanism of ISL against intestinal mucositis. Subsequent animal experiments showed that ISL could reduce the weight loss, leukopenia and mucosal damage caused by 5-FU. Compared with the intestinal mucositis model, the protein expressions of PTGS2, NOS2, TNFα (Tumor necrosis factor-alpha) and NF-κB p65 (nuclear factor kappa-B P65) were decreased after ISL treatment. In conclusion, this study is the fist time to find that ISL can attenuate 5-FU-induced intestinal mucositis in mice. Its anti-mucositis effect may be through regulating TNF/NF-κB pathway and inhibiting inflammatory mediators PTGS2 and NOS2. It will provide a potential candidate for the prevention and treatment of chemotherapy-induced intestinal mucositis.

## 1 Introduction

5-fluorouracil (5-FU) is an analogue of uracil (McQuade et al., 2016). It can be activated into fluorouracil deoxynucleotide (FdUMP) in cells, which disrupts DNA synthesis by suppressing thymidylic acid synthase (Panchenko et al., 2018). 5-FU has been the backbone of therapy for many solid tumors, such as esophageal, breast, stomach and intestinal malignant tumors (McQuade et al., 2016; Zhang et al., 2019).

However, the non-selective cytotoxicity of 5-FU can cause serious side effects, including gastrointestinal dysfunction, mucosal inflammation, cardiotoxicity, and even myelosuppression (Atiq et al., 2019). Mucositis is the most common gastrointestinal toxicity, involving the entire gastrointestinal tract from the mouth to the anus. Especially intestinal mucositis has been regarded as one of the main obstacles to tumor chemotherapy. Intestinal mucositis usually presents with anorexia, nausea, vomiting, diarrhea and so on (Justino et al., 2020). And severe mucositis will develop into ulcers that invade the entire submucosa. This can cause severe pain and even require narcotic analgesics to relieve it (Xing et al., 2015). So that, patients have to reduce the drug dose or discontinue the medication, which may lead to efficacy reduction or treatment failure. However, the clinical treatment of intestinal mucositis is mainly symptomatic support, such as drugs to relieve pain or diarrhea. So far, chemotherapy-induced intestinal mucositis is still lack of effective drugs to target its pathogenesis. Therefore, it is necessary to explore drugs for intestinal mucositis.

Traditional Chinese medicine (TCM) has the advantages of low adverse reactions and stable efficacy. It has been widely used in tumor adjuvant therapy to enhance the efficacy of chemotherapy and reduce toxic effects. Clinical studies have shown that TCM can significantly reduce the incidence of diarrhea and other gastrointestinal symptoms during chemotherapy. Among these TCM, licorice, called Gancao in Chinese, is the most frequently used herbal medicine (Yang et al., 2017). In TCM treatment, licorice is one of the essential Chinese herbal medicines for treating gastrointestinal diseases (Yang et al., 2017).

ISL is one of the most important bioactive compounds in licorice (Tang et al., 2018). Numerous studies have demonstrated that ISL has inhibitory effects on various malignant tumors, such as breast cancer, prostate cancer, colon cancer, lung cancer, Ovary Cancer, cervical cancer and leukemia (Wang et al., 2021). It can also be combined with chemotherapeutic agents to enhance anti-tumor efficacy (Peng et al., 2015). In addition, ISL is an effective anti-inflammatory agent, which can reduce inflammation by inhibiting IL-1β, IL-6, TNFα and other cytokines (Du et al., 2016; Liao et al., 2020). Recent studies have shown that ISL can significantly suppress the dextran sulfate sodium (DSS)-induced colitis-associated tumorigenesis in mice and maintain the integrity of the intestinal mucosal barrier (Zhao et al., 2014; Wu C. H. et al., 2016). Those findings suggest that ISL can protect intestinal mucosa and reduce inflammatory injury. It may be used as an adjunctive agent to relieve 5-FU-induced intestinal mucositis during chemotherapy. Although the antitumor and the synergistic effect of ISL have been widely reported, the protective effect of ISL on intestinal mucositis has not been investigated, which is also one of the major factors affecting cancer treatment.

Network pharmacology is a new branch of pharmacology based on system biology and computer science. By constructing the molecular network of a biological system, the overall relationship between drug-target-disease can be comprehensively analyzed, and the target and mechanism of the drug on diseases can also be predicted (Zhu et al., 2018). Nowadays, network analysis has been widely used to screen the active ingredients and explore the mechanism of TCM.

**FIGURE 1:**
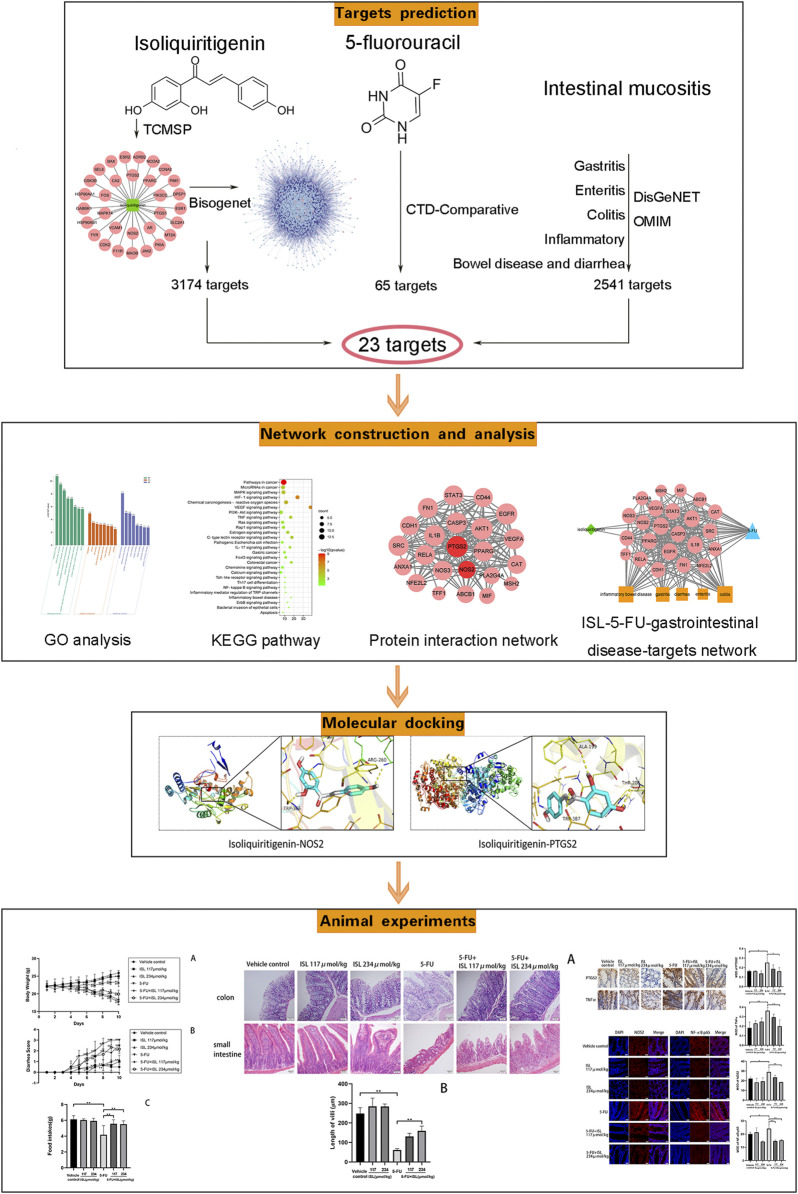
Flow chart of this study.

In this study, a comprehensive network analysis approach was used to predict the mechanism of ISL on 5-FU-induced gastrointestinal toxicity. And the prediction results were further verified in a mouse model of intestinal mucositis. The flow of this study was shown in Figure 1. This is the first time to explore the effect and mechanism of ISL against intestinal mucositis induced by 5-FU. It may provide reference for clinical treatment and drug development of chemotherapy-induced intestinal mucositis.

## 2 Materials and methods

### 2.1 Collection of pharmacological targets

The TCMSP database (https://old.tcmsp-e.com/tcmsp.php) (Fan et al., 2022) was employed to predict the pharmacological targets of ISL (ISL target, iT). A total of 31 iTs were collected. And the Bisogenet plug-in in Cytoscape 3.8.0 (Martin et al., 2010) was used to screen out 3,174 target proteins related to ISL targets (related target, rT), thus expanding the range of prediction targets and protein interaction network. The therapeutic targets for the gastrointestinal disease were obtained from the DisGeNET database (http://www.disgenet.org/) (Piñero et al., 2017) and the OMIM database (https://omim.org/) (Amberger et al., 2015). Five diseases associated with gastrointestinal injury were searched in those databases, such as gastritis, enteritis, colitis, inflammatory bowel disease and diarrhea. A total of 2,541 targets (duplicate value removed) associated with gastrointestinal disease (disease target, dT) were collected. Then, the CTD-Comparative database (http://ctdbase.org/) (Aihaiti et al., 2021) was used for the collection of 5-FU gastrointestinal toxic targets (toxic targets of 5-FU, tT). In total, 65 potential tTs were collected.

### 2.2 Network construction and analysis

23 common targets were found among iT, rT, tT and dT, and submitted to the DAVID database (https://david.ncifcrf.gov/) (Aihaiti et al., 2021) to use Gene ontology (GO) analysis and Kyoto Encyclopedia of Genes and Genomes (KEGG) pathway enrichment analysis. The protein interaction relationship of the common targets was obtained by the database STRING (https://www.string-db.org/) (Aihaiti et al., 2021). And the networks were visualized using Cytoscape software (http://www.cytoscape.org/) (Aihaiti et al., 2021).

### 2.3 Molecular docking

To verify the critical targets of ISL, a molecular docking of ISL with PTGS2 (Prostaglandin G/H synthase 2) and NOS2 (Nitric oxide synthase, inducible) was performed. And Known inhibitors of PTGS2 (Celecoxib) and NOS2 (N-Iminoethyl-L-lysine dihydrochloride) were used as reference (Alqahtani, 2021; Lawal et al., 2021). The 3D structures of PTGS2 and NOS2 were obtained from the Protein Data Bank (PDB, http://www.rcsb.org/) and then imported into PyMOL (v2.3.0) to remove water molecules. The structure of ISL, Celecoxib and N-Iminoethyl-L-lysine dihydrochloride were downloaded from the website of PubChem (https://pubchem.ncbi.nlm.nih.gov/) and then imported into PyMOL (v2.3.0) and AutoDockTools-1.5.6 software. The target proteins and ligands were prepared for molecular docking by Autodock Tools (ADT) program. Finally, molecular docking analysis was performed using the AutoDock Vina program (Trott and Olson, 2010). The crystal structures used for PTGS2 and NOS2 and the parameter settings for the grid box were provided in the Supplementary Material S1.

### 2.4 Pharmacological experimental validation of network analysis

#### 2.4.1 Materials

ISL (HPLC ≥98%) was purchased from Chengdu Pufei De Biotechnology Co., Ltd. 5-FU (HPLC >99%) was purchased from Dalian Meilun Biotechnology Co., Ltd. ISL was dissolved in saline containing 2% dimethylsulfoxide (DMSO). 5-FU was prepared in pyrogen-free saline.

### 2.4.2 Animals and treatment

36 male SPF BLAB/c mice (18–20 g, 6–8 weeks old) were obtained from Chengdu Dossy Experimental Animals Co., Ltd. Mice were raised under standard laboratory conditions at 23 ± 1°C and 55 ± 5% humidity with a 12 h light/dark cycle.

**FIGURE 2:**
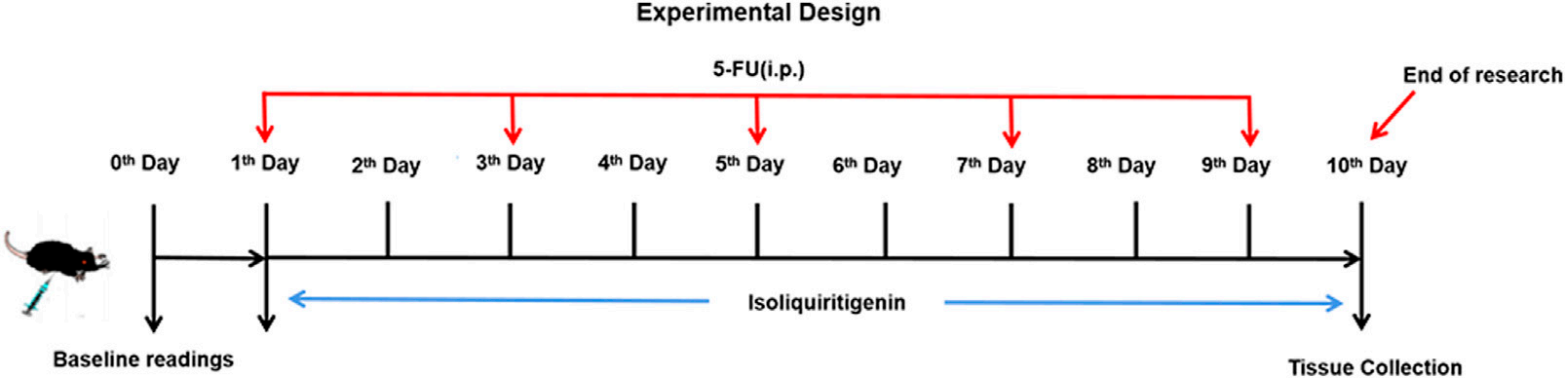
Experimental protocol for inducing intestinal mucositis in mice.

The mice were randomly allocated to six groups (6 mice per group): 1) Vehicle control (2% DMSO in saline); 2) ISL 117 μmol/kg; 3) ISL 234 μmol/kg; 4) Model (5-FU 384 μmol/kg); 5) 5-FU + ISL 117 μmol/kg; 6) 5-FU + ISL 234 μmol/kg. All drugs were injected intraperitoneally (i.p.). Except for the vehicle control and model (5-FU) group, all mice received ISL for 10 days. Mice in model and 5-FU + ISL groups were intraperitoneally injected with 5-FU (384 μmol/kg) every other day. The treatment schedule was shown in Figure 2. All mice were fed and waterad libitum during the entire experiment.

All animal experiments were approved by the Experimental Animal Ethical Committee of Chengdu Medical College, and the experimental protocols were carried out strictly in conformity with the guidance for Animal Experiments in Chengdu Medical College and the Declaration of Helsinki.

#### 2.4.3 The routine observation (body weight, food intake and diarrhea) in mice

Mice were monitored daily for body weight, food intake and diarrhea. Bowen’s score system was used to assess diarrhea severity, and the grade ranged from 0 to 3 based on the consistency of stool: 0, normal stool; 1, the stool is slightly wet or soft, indicating mild diarrhea; 2, the stool is moderately wet with unformed pellets, indicating moderate diarrhea; 3, the stool is watery or loose stool, indicating severe diarrhea (Yeung et al., 2015; Atiq et al., 2019).

### 2.4.4 Sample collection and preparation

24 h after the last administration, the mice were anesthetized by intraperitoneal injection of 2% pentobarbital sodium. Then, 1 ml Peripheral blood from each mouse was collected *via* orbital blood and placed into an anticoagulant tube containing EDTA. The blood was prepared for a routine blood test, which included the analysis of white blood cell (WBC) count, neutrophil count, lymphocyte count, red blood cell (RBC) count and so on. All mice were then sacrificed immediately. And 1–2 cm of the middle segment of the small intestine and colon was dissected, washed with saline, and then fixed with paraformaldehyde for further analysis.

### 2.4.5 Histopathological analysis

The paraformaldehyde-fixed small intestine and colon tissues were embedded in paraffin and sectioned at 3 μm thickness. The paraffin sections were baked at 60°C for 3 h. Then, sections were dewaxed with xylene, and placed in gradient ethanol (absolute ethanol twice for 3 min, 95% ethanol for 3 min, 75% ethanol for 3 min), and finally stained with hematoxylin for 12 min and eosin for 13 s 3-5 complete longitudinal small intestinal villi were selected from each sample for measurement (3 samples per group). The villi height was measured from the top of the villus to the villus-crypt junction using ImageJ software (Atiq et al., 2019).

#### 2.4.6 Immunohistochemistry and immunofluorescence experiments

After dewaxing and rehydration, paraffin sections of colon tissue were placed in sodium citrate buffer and heated by a microwave oven twice for 2–3 min to repair antigens. After washing twice with PBS for 5 min, 5% bovine serum albumin (BSA) was added to block non-special signals for 30 min at 37°C. BSA was then removed, and primary antibodies (1:200, Proteintech Group, Inc, United States) diluted with 1% BSA were added and incubated overnight at 4°C. After washing with PBS, slides were incubated with horseradish peroxidase (HRP)- or Alexa Fluor-secondary antibodies (1:1,000, Proteintech Group, Inc, United States) at room temperature in dark for 1 h. The slides were washed twice with PBS for 5 min, and incubated with diaminobenzidine (DAB) for 3–10 min to develop color, or with DAPI for 10 min to stain nuclei. Finally, the slides were sealed by neutral glue or anti-fluorescence quencher. 3-5 fields were randomly selected for each sample (3 samples per group) and observed under an Olympus BX63 fluorescence microscope (Olympus, Tokyo, Japan) at ×400. And ImageJ analysis software was used to assess the staining results. Protein expression was quantified as mean optical density (MOD = integrated option density/area). (Du et al., 2015; Wang et al., 2022).

### 2.4.7 Statistics

Quantitative data were represented as the means ± standard deviations (SD). SPSS and GraphPad Prism were used for statistical analysis and graph drawing, and the differences between groups were analyzed by one-way analysis of variance (ANOVA). *p* < 0.05 was considered statistically significant.

## 3 Results

### 3.1 Target prediction of ISL against 5-FU-induced gastrointestinal toxicity

**FIGURE 3:**
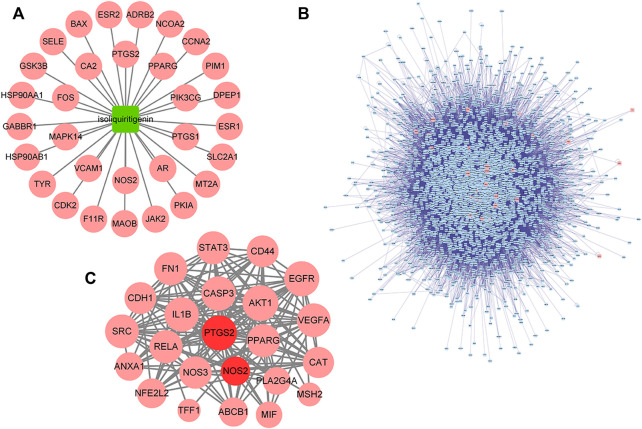
Targets of ISL against 5-FU gastrointestinal toxicity predicted by network analysis. **(A)** The ISL-target network. The node in green color is ISL, and the nodes in red color are candidate targets of ISL. **(B)** Protein interaction network of ISL target (iT) and its related target (rT). The nodes in red color are iTs, and the nodes in blue color are rTs. **(C)** Protein interaction network of potential targets (pT) of ISL for detoxification. The nodes in red color are pTs for ISL against the gastrointestinal toxicity of 5-FU, the nodes in dark red color are the critical targets for the detoxification of ISL.

Based on target prediction *via* TCMSP, the ISL-target network (Figure 3A) consisted of 31 nodes. To better evaluate the detoxification effect of ISL on 5-FU, rTs interacting with iTs were screened to expand the range of predicted targets, and a total of 3,174 rTs were obtained (Figure 3B). Finally, 23 common targets were obtained from the intersection of tTs, dTs and iTs or rTs. The 23 targets could be considered as potential targets (pT) for ISL to reduce the gastrointestinal toxicity of 5-FU. Among them, PPARG, PTGS2 and NOS2 were the direct targets of ISL (iT) predicted by TCMSP. Moreover, PTGS2 (also known as cyclooxygenases-2, COX-2) and NOS2 (also known as inducible nitric oxidesynthase, iNOS) have been reported to be involved in the occurrence of 5-FU-caused intestinal mucositis (Atiq et al., 2019; Yan et al., 2020; Huang et al., 2022). Therefore, PTGS2 and NOS2 could be considered as critical targets of ISL against 5-FU-induced gastrointestinal toxicity.

### 3.2 Network analysis on the mechanism of ISL against 5-FU-induced gastrointestinal toxicity

**FIGURE 4:**
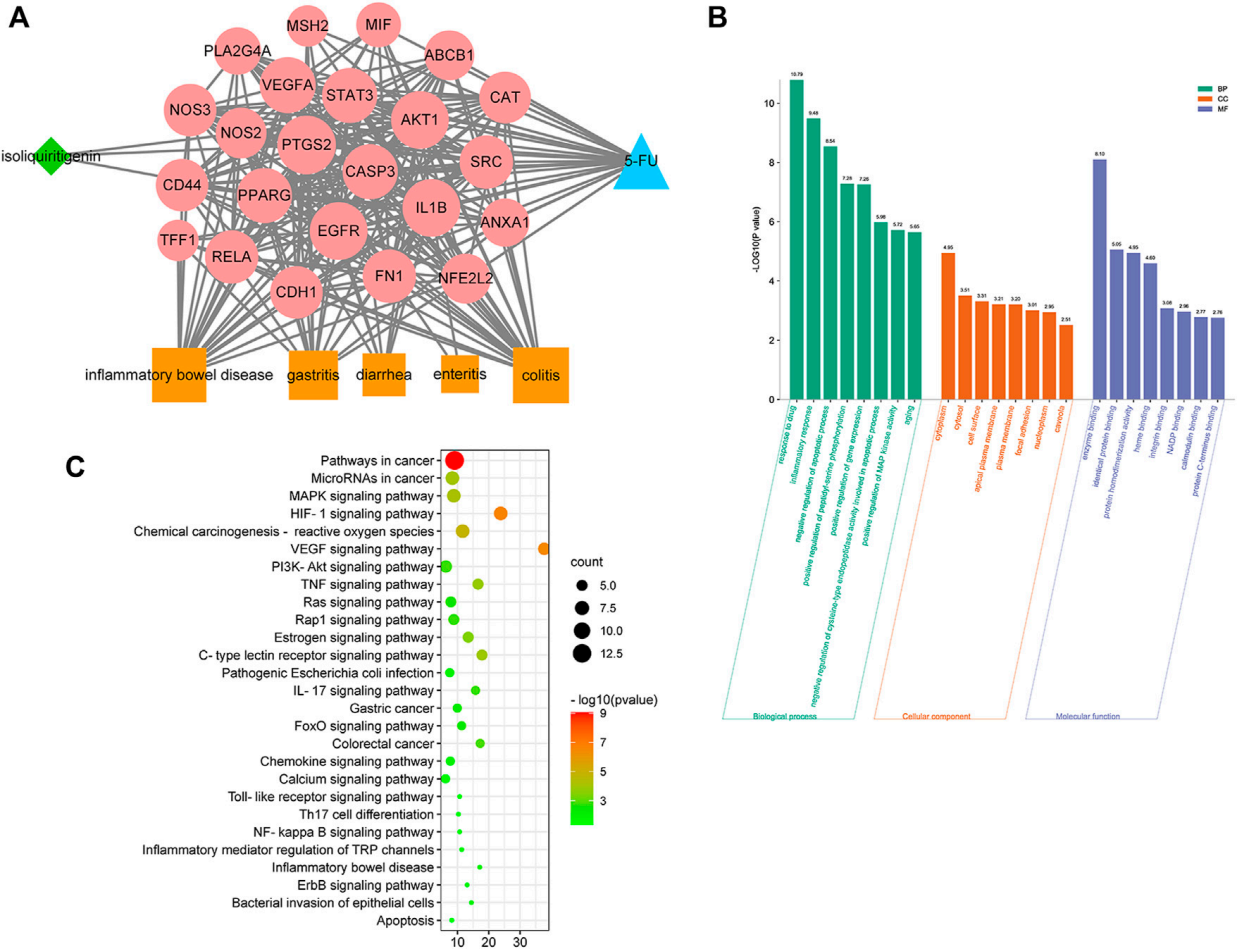
Network analysis on the mechanism of ISL against 5-FU-induced gastrointestinal toxicity. **(A)** Network of ISL, potential targets, 5-FU, and gastrointestinal diseases (ISL-pT-5FU-D Network). The node in blue color is 5-FU, the nodes in red color are potential targets, the nodes in orange color are gastrointestinal diseases, and the node in green color is ISL. **(B)** KEGG pathway enrichment analysis of potential targets. The pathways related to inflammation and gastrointestinal diseases with *p* values less than 0.05 are shown. **(C)** GO enrichment analysis of potential targets: cellular components, molecular functions, and biological processes associated with the detoxification effect of ISL on 5-FU. The top 8 with lower *p*-value are shown.

As shown in Figure 4A, a network of ISL-potential targets-5-FU-gastrointestinal disease network (ISL-pT-5FU-D) was constructed to reflect the relationship between ISL (green nodes), 5-FU (blue nodes), gastrointestinal diseases (orange nodes) and their common targets (red nodes). We further investigated the correlation degree of each node and sequenced them with the node size (Shao, 2021). More information about the nodes was provided in the Supplementary Materials S2.

Next, a bioinformatics analysis of potential targets was performed. As shown in Figures 4B,C, the alleviating effect of ISL on 5-FU-induced gastrointestinal toxicity might be related to biological processes such as inflammatory response, apoptosis regulation, NO production and NF-kappa B transcription factor activity. And the molecular mechanism might involve multiple signaling pathways, such as HIF-1, VEGF, TNF, MAPK, IL-17, PI3K-Akt, Ras, NF-kappa B, Toll-like receptor pathway. Therefore, network analysis suggested that ISL might alleviate 5-FU-induced gastrointestinal toxicity through multiple targets and pathways, which were mainly involved in regulating inflammation and apoptosis.

### 3.3 Molecular docking validation of the predicted critical targets

**FIGURE 5:**
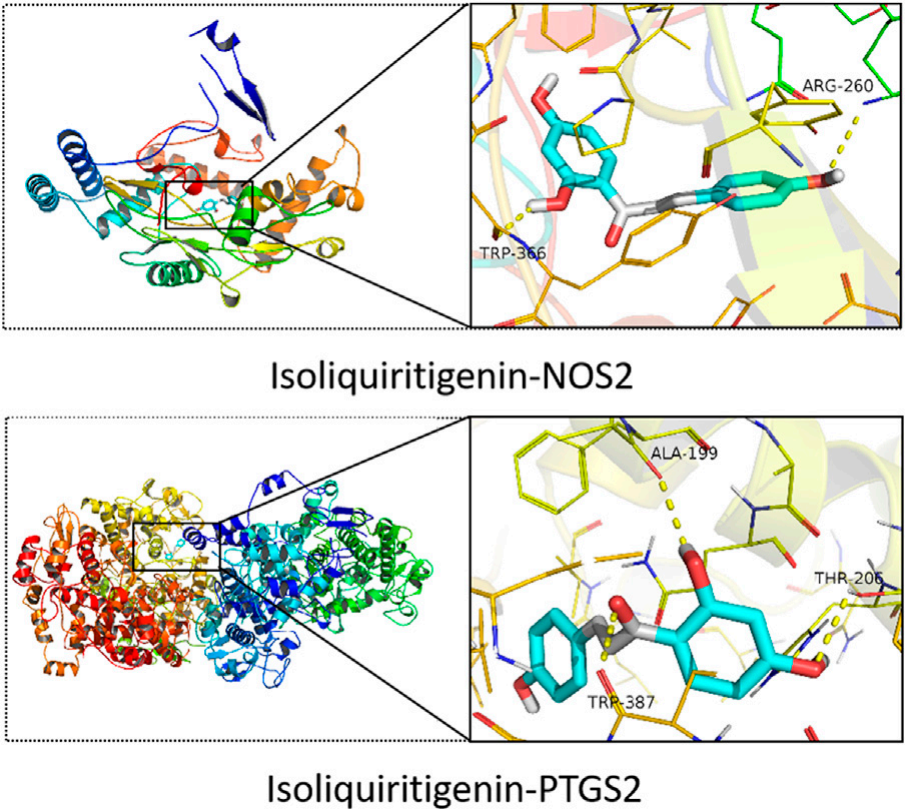
Molecular docking of ISL with PTGS2 and NOS2.

To verify the accuracy of the above predictions, a molecular docking analysis of ISL with PTGS2 and NOS2 enzyme was performed. ISL has large binding affinity for PTGS2 and NOS2 protein with binding energies of −9.1 and −8.7 kcal/mol, respectively. Furthermore, ISL showed a higher binding energy with PTGS2 and NOS2 than the known protein inhibitor (Table 1). This suggested that ISL might bind and inhibit PTGS2 and NOS2 protein. In addition, as shown in Figure 5, ISL can interact with NOS2 by two H-bonds with ARG260 and TRP366, and interact with PTGS2 by three H-bonds with TRP387, THR206 and ALA199. ISL was successfully docked with PTGS2 and NOS2 protein.

**TABLE 1:** The binding energies of ISL with NOS2 and PTGS2 protein targets.

Protein	PDB ID	Binding energy (kcal/mol)	
		Inhibitor	ISL
NOS2	2BHJ	−6.1	−9.1
PTGS2	1CX2	−8.1	−8.7

NOS2 Inhibitor: N-Iminoethyl-L-lysine dihydrochloride.

PTGS2 inhibitor: Celecoxib.

### 3.4 Effect of ISL on body weight, diarrhea, food intake and blood routine index in mice model

**FIGURE 6:**
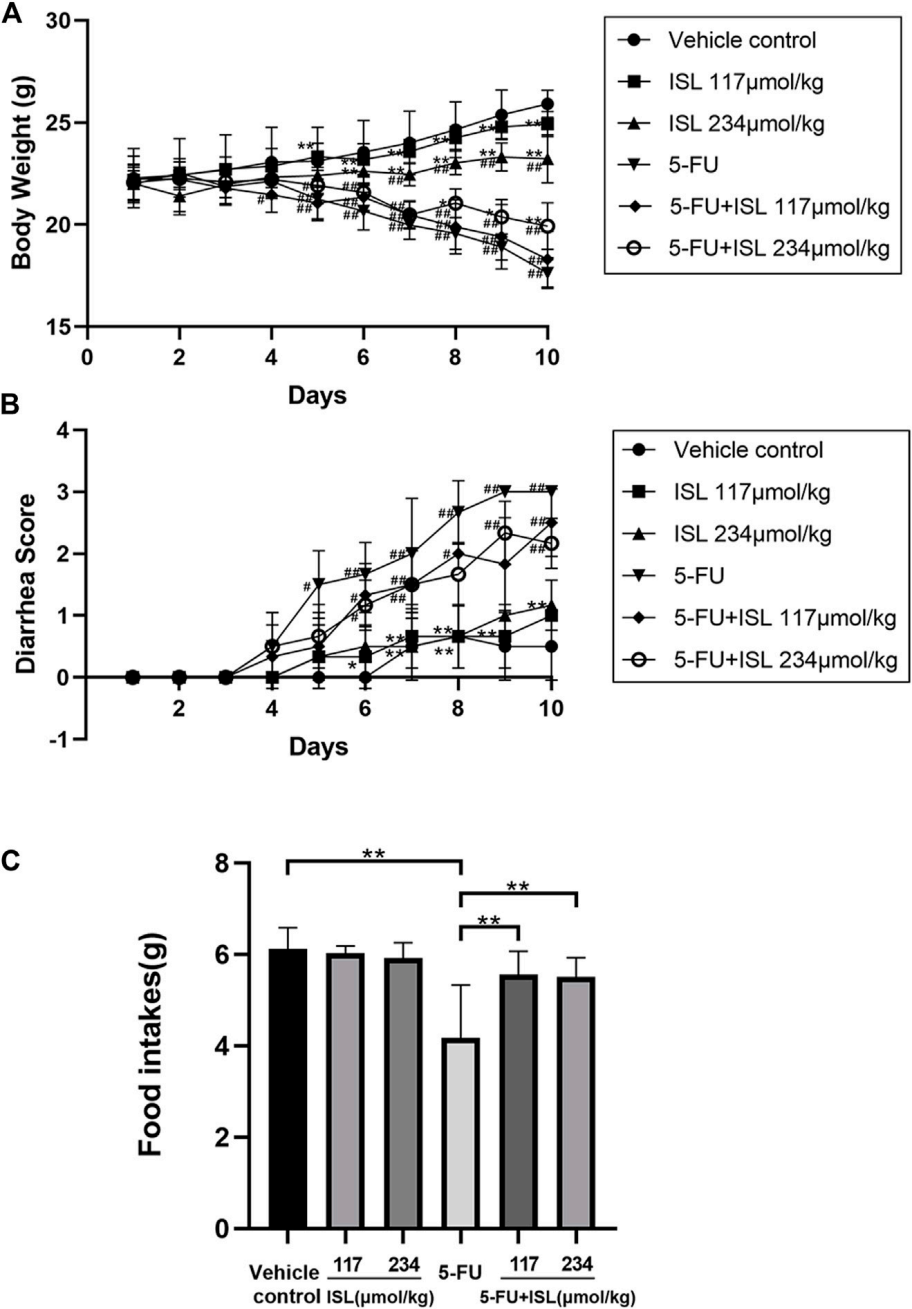
Effect of ISL on body weight, diarrhea, and daily food intake in mice with or without 5-FU intraperitoneal injection. **(A)** Changes in daily body weights. **(B)** Changes in daily diarrhea. **(C)** Mean daily food intake. Data are presented as mean ± S.D. (n = 6 in each group). #*p* < *0.05* and ^##^ *p* < *0.01* vs. control group; *p < *0.05* and **p < *0.01* vs. 5-FU group.

To assess the severity of gastrointestinal injury caused by 5-FU, mice were monitored daily for body weight, food intake and diarrhea. The results were shown in Figure 6. Compared with the vehicle control, intraperitoneal injection with 5-FU significantly reduced body weight, increased diarrhea, and decreased food intake of mice (*p* < 0.05). After combined treatment with ISL, the weight loss of mice was slower, and the food intake in mice was increased compared with that of the 5-FU group (*p* < 0.05). Although no significant difference was observed in diarrhea scores between the 5-FU and 5-FU + ISL groups, the diarrhea of mice in the combination groups showed a decreasing trend.

Furthermore, the blood routine indexes were detected to evaluate the effect of ISL on hematotoxicity. As shown in Table 2, the counts of WBC, lymphocytes, neutrophils and RBC were significantly decreased in the 5-FU group compared with the vehicle control group (*p* < 0.05). And compared to the 5-FU group, the WBC, lymphocytes, and neutrophils indexes in the 5-FU + 234 μmol/kg ISL combination group were significantly increased (*p* < 0.05). Thus, these results suggested that ISL could alleviate gastrointestinal toxicity symptoms and leukopenia caused by 5-FU.

**TABLE 2:** Changes in indexes of blood routine in mice (n = 3 in each group).

Groups	WBC(10^9^/L)	LY (10^9^/L)	NE (10^9^/L)	RBC(10^12/^L)
Control	5.07 ± 0.49	4.30 ± 0.26	0.70 ± 0.17	10.15 ± 0.14
5-FU	1.00 ± 0.36^##^	0.67 ± 0.25^##^	0.30 ± 0.10^#^	9.21 ± 0.21^#^
5-FU + ISL (117 μmol/kg)	0.97 ± 0.29^##^	0.70 ± 0.20^##^	0.23 ± 0.12^##^	9.62 ± 0.69
5-FU + ISL (234 μmol/kg)	2.13 ± 0.49^##/*^	1.40 ± 0.17^##/**^	0.67 ± 0.0.21^*^	9.57 ± 0.17

WBC, white blood cell count; LY, lymphocyte count; NE, neutrophil count; RBC, red blood cell count. Data are presented as mean ± S.D. ^#^ *p* < *0.05* and ^##^ *p* < *0.01* vs. control group; *p < *0.05* and **p < *0.01* vs. 5-FU, group.

### 3.5 ISL alleviates mucosal damage induced by 5-FU in mice

**FIGURE 7:**
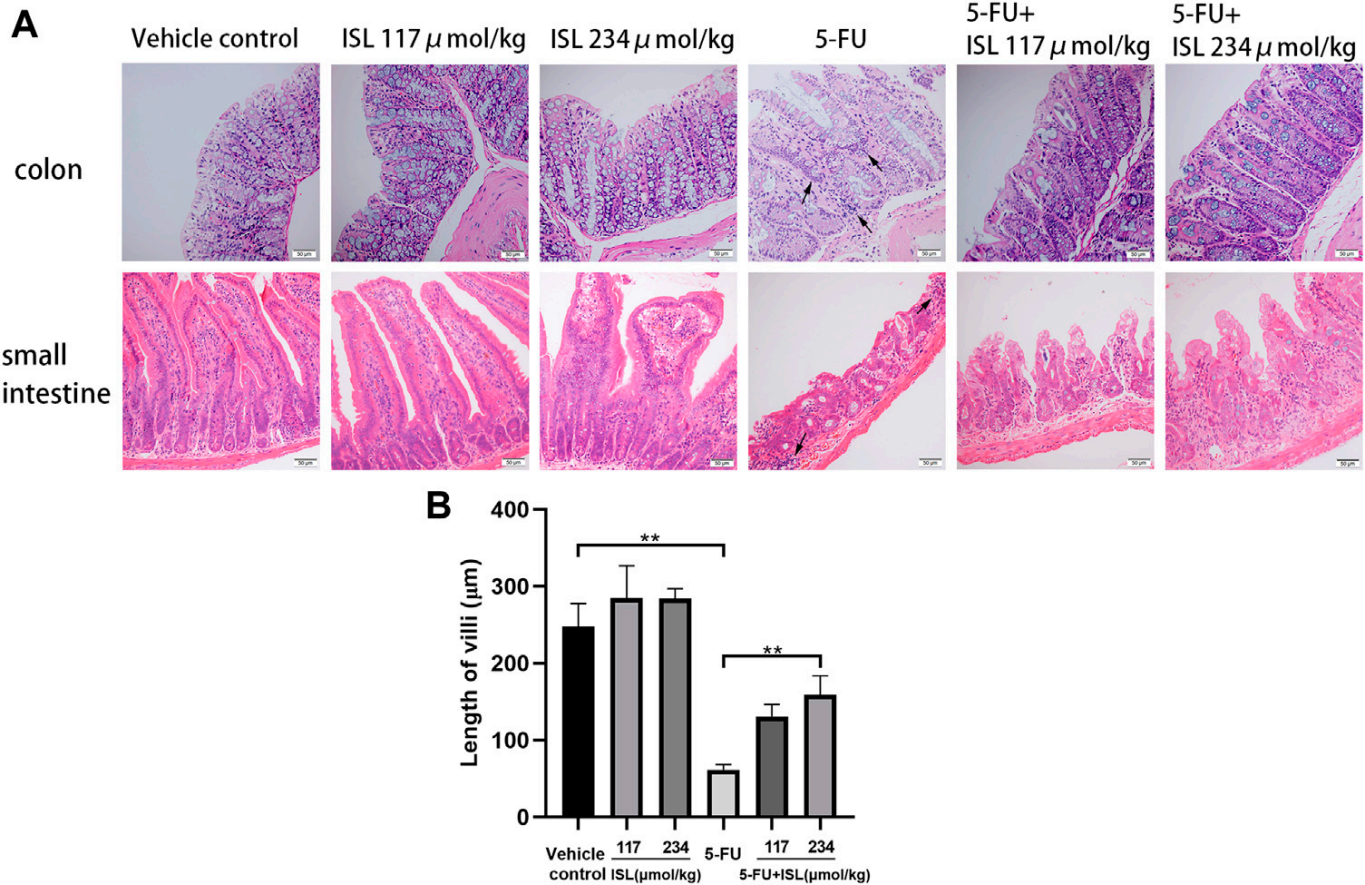
Effect of ISL on intestinal histopathological damage in mice. **(A)**Hematoxylin and eosin (H&E) staining of small intestine and colon. All images are displayed at ×200 magnification. Inflammatory cell infiltration (black arrows) is observed in the 5-FU group. **(B)** Length of small intestinal villi. Data are presented as mean ± S.D. (n = 3 in each group). **p < 0.01.

As shown in Figure 7, no histomorphological changes were observed in the small intestine and colon of the vehicle control mice, with clear intestinal tissue structure, neatly arranged villi, normal epithelial cell morphology, and densely arranged intestinal glands. In the ISL groups, the mucosal structure of mice remained intact. The intestinal mucosa of mice in the 5-FU group was the most seriously damaged, with loose cell arrangement and obvious inflammatory cell infiltration. And the villi heights were significantly lower than that of the vehicle control (*p* < 0.01). Compared with the 5-FU group, the mice in the 5-FU + ISL combination groups exhibited less mucosal damage, less inflammatory cell infiltration, and greater villi height (*p* < 0.01). It indicated the protective effect of ISL on 5-FU-induced intestinal mucosal damage.

### 3.6 The effects of ISL on the expressions of PTGS2, NOS2, TNFα and NF-κB p65

**FIGURE 8:**
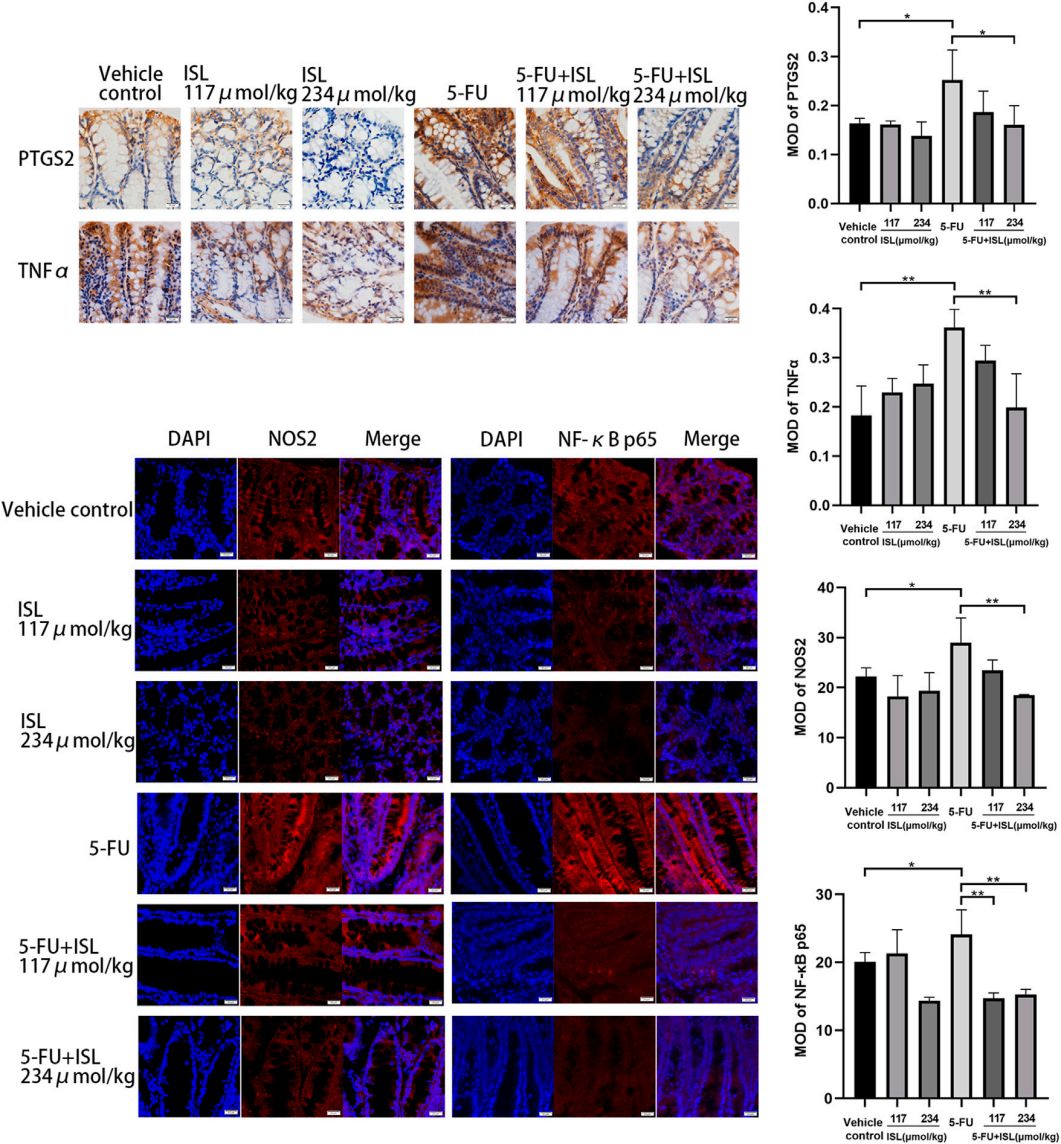
Immunohistochemistry and immunofluorescence analysis for PTGS2, NOS2, TNFα and NF-κB p65. MOD, mean optical density. All images are displayed at ×400 magnification. Data are presented as mean ± SD. (n = 3 in each group). *p < 0.05 and **p < 0.01.

To verify the molecular mechanism of ISL against 5-FU-induced intestinal mucositis, the expression of PTGS2, NOS2, TNFα, and NF-κB p65 in colon tissues were detected. As shown in Figure 8, compared with the vehicle control, 5-FU treatment significantly increased the expression of PTGS2, NOS2, TNFα and NF-κB p65 (*p* < 0.05). These protein levels were obviously decreased in the colon tissues of mice in the 5-FU + ISL combination groups compared to the 5-FU group. And the decrease in the 5-FU+234 μmol/kg ISL group was highly significant (*p* < 0.05). The results suggested that the alleviating effect of ISL on 5-FU-induced intestinal mucositis might be related to the inhibition of PTGS2, NOS2, TNFα and NF-κB p65, which was consistent with our network pharmacological predictions.

## 4 Discussion

Currently, 5-FU is a routine treatment for a variety of malignant tumors. It is also known to cause severe gastrointestinal toxicity, such as intestinal mucositis. In intestinal mucositis, 5-FU can induce cell apoptosis, force intestinal mucosa to atrophy, damage the intestinal villi, and reduce the absorption of intestinal contents and water, thus causing dyspepsia, anorexia, abdominal pain and diarrhea (Yan et al., 2020). These serious toxic effects of 5-FU will reduce patients’ treatment compliance, and even lead to the interruption of chemotherapy (Kato et al., 2015; Xing et al., 2015). Therefore, it is necessary to explore novel drugs to prevent and treat intestinal mucositis caused by 5-FU.

Recent studies have shown that inflammatory cytokines IL-1β, IL-6, TNFα, and NF-κB are significantly increased in 5-FU-induced intestinal mucositis in mice (Ali et al., 2019; Atiq et al., 2019). 5-FU can induce the production of reactive oxygen species (ROS), promote DNA damage and then activate NF-κB (Chang et al., 2012; Oliveira et al., 2021). Next, NF-κB activation leads to the release of inflammatory mediators, including TNFα, COX-2, iNOS, IL-1β, and so on (Kim et al., 2009; Abdelhamid et al., 2020). TNFα, in turn, activates NF-κB. This positive feedback mechanism results in signal amplification and increased production of inflammatory mediators, which eventually destroy the intestinal mucosal barrier and lead to the formation of mucosal ulcers (Huang et al., 2022; Shi et al., 2022).

In this study, a mouse model of intestinal mucositis was established by intraperitoneal injection of 5-FU. Consistent with the previous studies, administration of 5-FU caused severe diarrhea, weight loss and anorexia in mice, which were the main symptoms of intestinal mucositis. (Soares et al., 2013; Zhang et al., 2019). And histopathological changes were also demonstrated, such as shortening of villi, inflammatory cell infiltration and loss of mucosal structure. And the expressions of NOS2, TNFα, NF-κB P65 and PTGS2 in colon tissues were significantly increased. It suggested that these inflammatory mediators and their signaling pathways might be implicated in the occurrence of intestinal mucositis.

ISL, a natural chalcone flavonoid, has been shown to exhibit good pharmacokinetic properties and a variety of biological activities (Wang et al., 2021), including anti-tumor, anti-inflammatory, immunomodulatory, gastrointestinal protection and so on (Wu M. et al., 2016; Zhao et al., 2019; Liao et al., 2020). In particular, its high antitumor effiacy has attracted extensive attention in recent years. Numerous studies have found that ISL can inhibit the proliferation and induce apoptosis of various cancer cells (Peng et al., 2015; Wang et al., 2021). At the same time, its cytotoxic effect shows selectivity, which can significantly inhibit the viability of tumor cells but almost no cytotoxicity to normal cells (Wu C. H. et al., 2016). ISL also significantly suppresses the growth and metastasis of various tumors in mouse models (Peng et al., 2015; Wang et al., 2021). In addition, ISL has synergistic antitumor effect with chemotherapies. For example, Zhang et al. reported that ISL synergistically sensitized the cytotoxic effect of gemcitabine and 5-FU on pancreatic cancer cells (Zhang et al., 2022). Consistently, Jin et al. demonstrated that ISL potentiated the apoptotic effects of 5-FU on HCT-116 cells (Jin et al., 2018). Overall, both *in vitro* and *in vivo* studies have demonstrated the potential of ISL for cancer treatment and adjuvant chemotherapy.

In addition, the anti-inflammatory effect of ISL has also been widely reported. Studies have shown that ISL can attenuate the inflammatory injury induced by LPS or carrageenan. Its anti-inflammatory effect is through inhibiting NF-κB and then causing iNOS, TNFα, COX-2, IL-6 and other inflammatory cytokines to down-regulation (Kim et al., 2008; Kim et al., 2008; Tang et al., 2018; Liao et al., 2020). Furthermore, ISL can protect mouse gastrointestinal tracts, and suppress DSS/AOM-induced colon inflammation-related tumorigenesis by down regulating PGE2 and IL-6 (Zhao et al., 2014; Wu M. et al., 2016). These findings suggest that ISL is a promising anti-inflammatory and intestinal protective agent. However, the effect of ISL on intestinal mucositis remains unknown.

Therefore, we observed the alleviating effect of ISL on 5-FU-induced intestinal mucositis in this study. The results showed that ISL could significantly reduce intestinal mucosal damage and its symptoms caused by 5-FU in mice. In addition, leukopenia, one of the main toxic effects of 5-FU, was also observed to be attenuated by ISL. These results reveal for the first time that ISL has good potential for alleviating 5-FU-induced gastrointestinal toxicity in adjuvant tumor therapy, and its mechanism is worthy of further investigation.

Network pharmacological analysis is a new approach to predict the potential pharmacological effects and mechanisms of TCM and its monomers. In this study, network analysis was used to predict the therapeutic targets of ISL on 5-FU-induced intestinal mucositis. And the results showed that PTGS2 and NOS2 might be the critical targets of ISL for reducing intestinal mucositis. In addition, KEGG and GO enrichment analysis indicated that the anti-intestinal mucositis mechanism of ISL was mainly related to inflammatory and apoptosis-related signaling pathways and biological processes, such as HIF-1 pathway, TNF pathway, PI3K-Akt pathway, NF-κB pathway, NO production, NF-κB transcription factor activity, *etc.*

**FIGURE 9:**
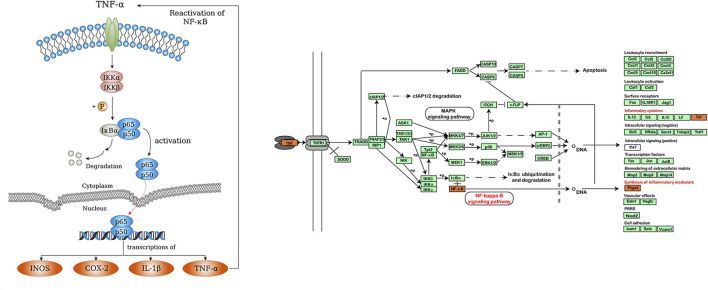
NF-κB and TNF signaling pathway.

As we know, TNF is the critical signaling pathway in regulating inflammatory responses (Chu, 2013). TNF family cytokines can promote the expression of inflammation-related genes mainly by activating the NF-κB pathway. (Hayden and Ghosh, 2011; Gilmore and Wolenski, 2012). NF-κB plays a critical role in the transcription of multiple inflammatory mediators. It is usually inactive in the form of p50/P65 dimer binding to its inhibitor kappaB (IκB) (Oeckinghaus et al., 2011). NF-κB can be activated by a variety of intracellular and extracellular stimuli, such as viruses, oxygen free radicals, and various cytokines (Lawrence, 2009; Oeckinghaus and Ghosh, 2009). Among them, TNF family cytokines are considered to be the best-characterized inducers of NF-κB (Ciuffa et al., 2017). Upon stimulation, IκB kinase is activated, resulting in the degradation of IκB protein and the release of NF-κB dimers. NF-κB dimers are then transferred into the nucleus to facilitate transcription of the target genes, such as iNOS, TNFα, COX-2 and IL-1β (Surh et al., 2001; Kim et al., 2009; Abdelhamid et al., 2020; Yu et al., 2020). Some of these inflammatory factors can bind to cell membrane receptors and reactivate NF-κB signaling (Figure 9).

Previous studies have suggested that TNF/NF-κB pathway and its downstream inflammatory mediators may be effective targets for alleviating chemotherapy-induced mucositis. For example, Yuan et al. reported that inhibiting NF-κB activity could reduce inflammation and mucosal damage caused by 5-FU in rats (Yuan et al., 2015). Ribeiro et al. demonstrated that down-regulation of NF-κB p65 and TNF attenuated 5-FU-induced oral mucositis (Ribeiro et al., 2017). Similarly, Barbosa et al. reported that suppressing inflammatory mediators, such as TNFα, IL-1 β and iNOS, could reduce oral mucositis (Barbosa et al., 2019). Moreover, Chang et al. found that COX-2 activity was increased in 5-FU-induced mucositis (Chang et al., 2012). In this study, we examined the effect of ISL on 5-FU-induced expression of TNFα, NF-κB, PTGS2 and NOS2 in mice with intestinal mucositis for the first time. PTGS2 (COX-2) and NOS2 (iNOS) were the critical targets of ISL predicted by network analysis. TNFα and NF-κB p65 were the key nodes in the TNF/NF-κB inflammatory pathway. The results showed that ISL significantly reduced the 5-FU-induced expression of TNFα, NF-κB, PTGS2 and NOS2 in colon tissues. It is consistent with the target prediction and KEGG pathway enrichment results of network pharmacological analysis (Figure 9).

## 5 Conclusion

In conclusion, the network analysis and pharmacological experimental verification show that ISL can ameliorate 5-FU-induced intestinal mucositis. Its mechanism may be related to the negative regulation of the TNF/NF-κB pathway, and the inhibition of inflammatory mediators PTGS2 and NOS2. Our study may provide a potential drug candidate for 5-FU-induced intestinal mucositis. Moreover, This study is the first to show that ISL alleviates the gastrointestinal toxicity of 5-FU, which will provide a new reference for the research and development of ISL. However, this study also has limitations, such as the mechanism of ISL against intestinal mucositis has not been thoroughly explored. And further *in vivo* and *in vitro* experiments will be considered in our future studies.

## Data Availability

The raw data supporting the conclusions of this article will be made available by the authors, without undue reservation.
