# Supplementary material for: Network analysis and experimental pharmacology study explore the protective effects of Isoliquiritigenin on 5-fluorouracil-Induced intestinal mucositis
Source: Front Pharmacol. 2022 Oct 6;13:1014160. doi: 10.3389/fphar.2022.1014160 (PMC9582754; doi:10.3389/fphar.2022.1014160)
Supplement: Supplementary file 1 [file DataSheet2.PDF]

Fig.8 (Results:Regulation of ISL on PTGS2, NOS2, TNFα and NF-κB p65)

The original images acquired under an fluorescence microscope at 400× magnification as follows.

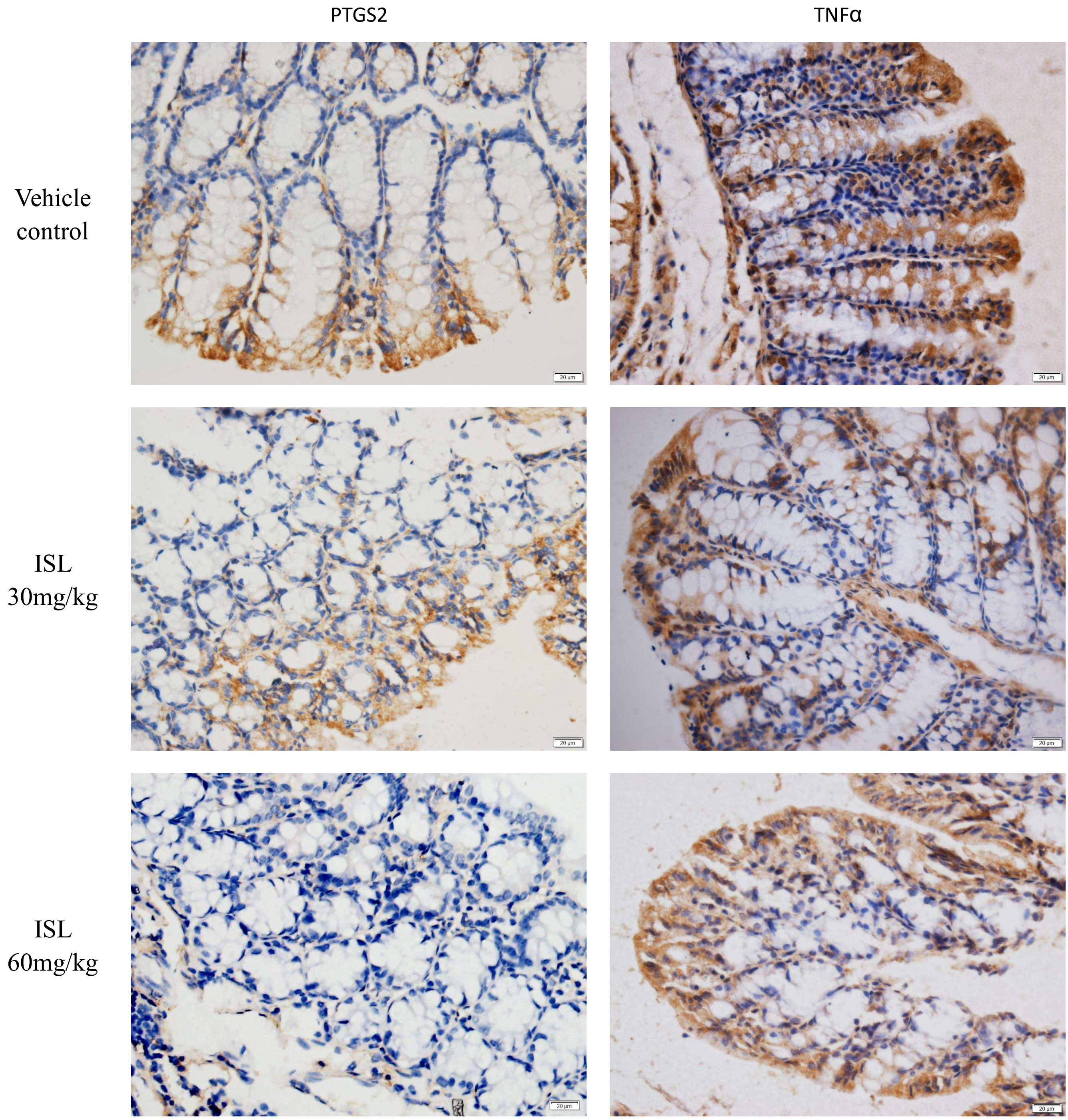

5-FU

5-FU+ISL
30mg/kg

5-FU+ISL
60mg/kg

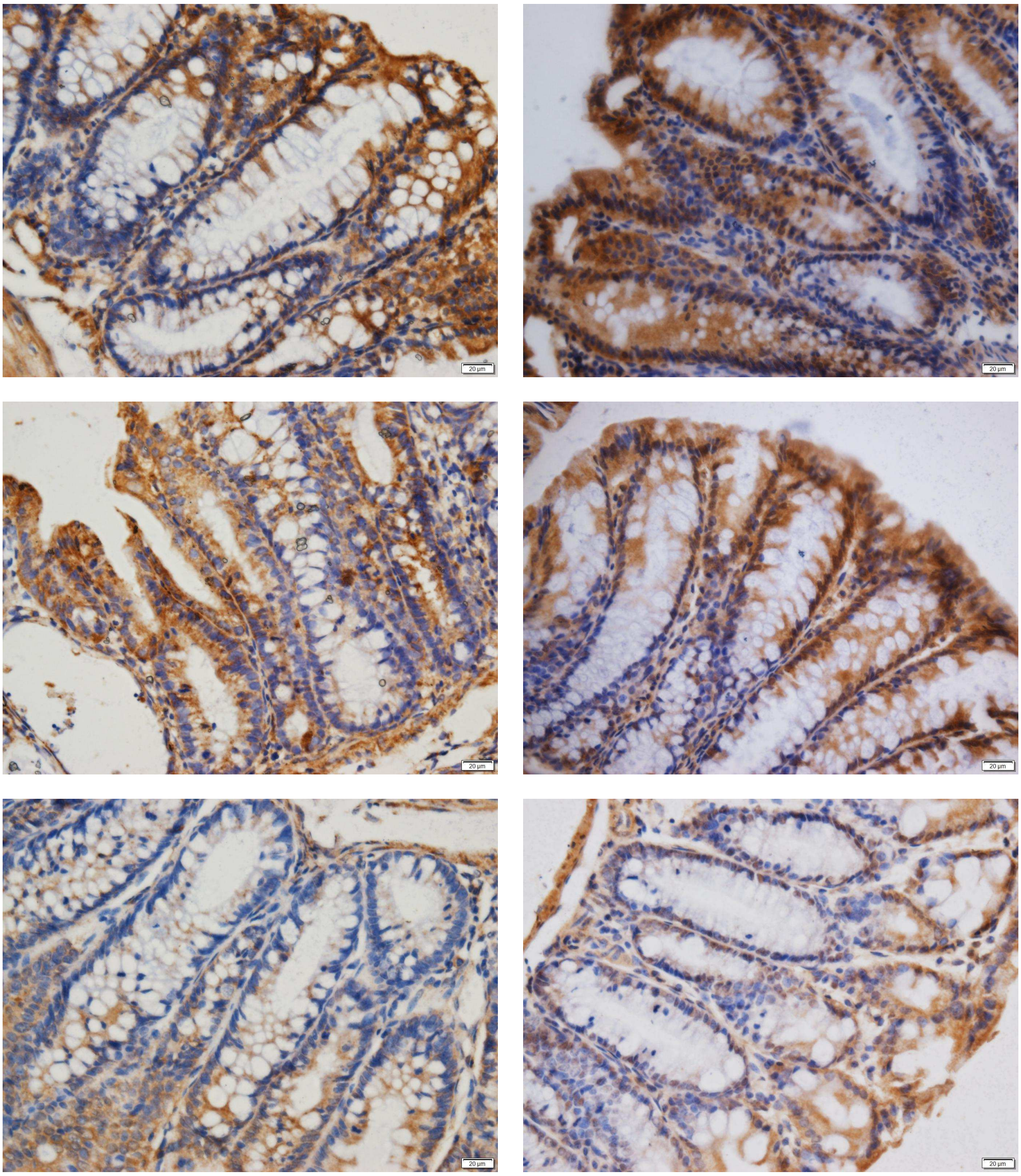

DAPI NOS2 Merge

Vehicle
control

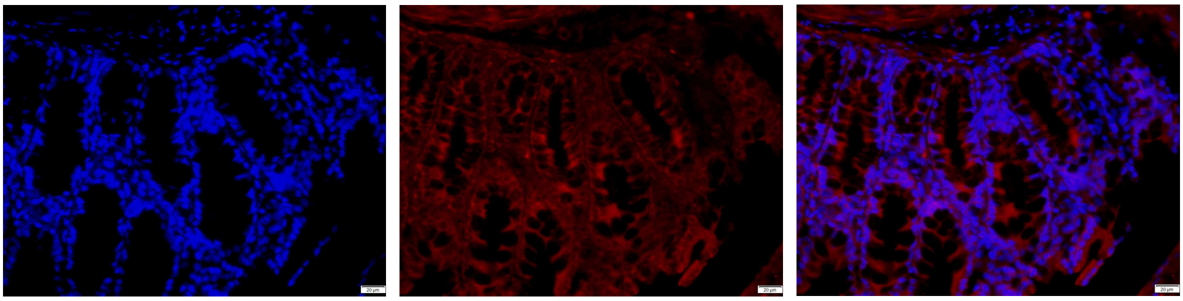

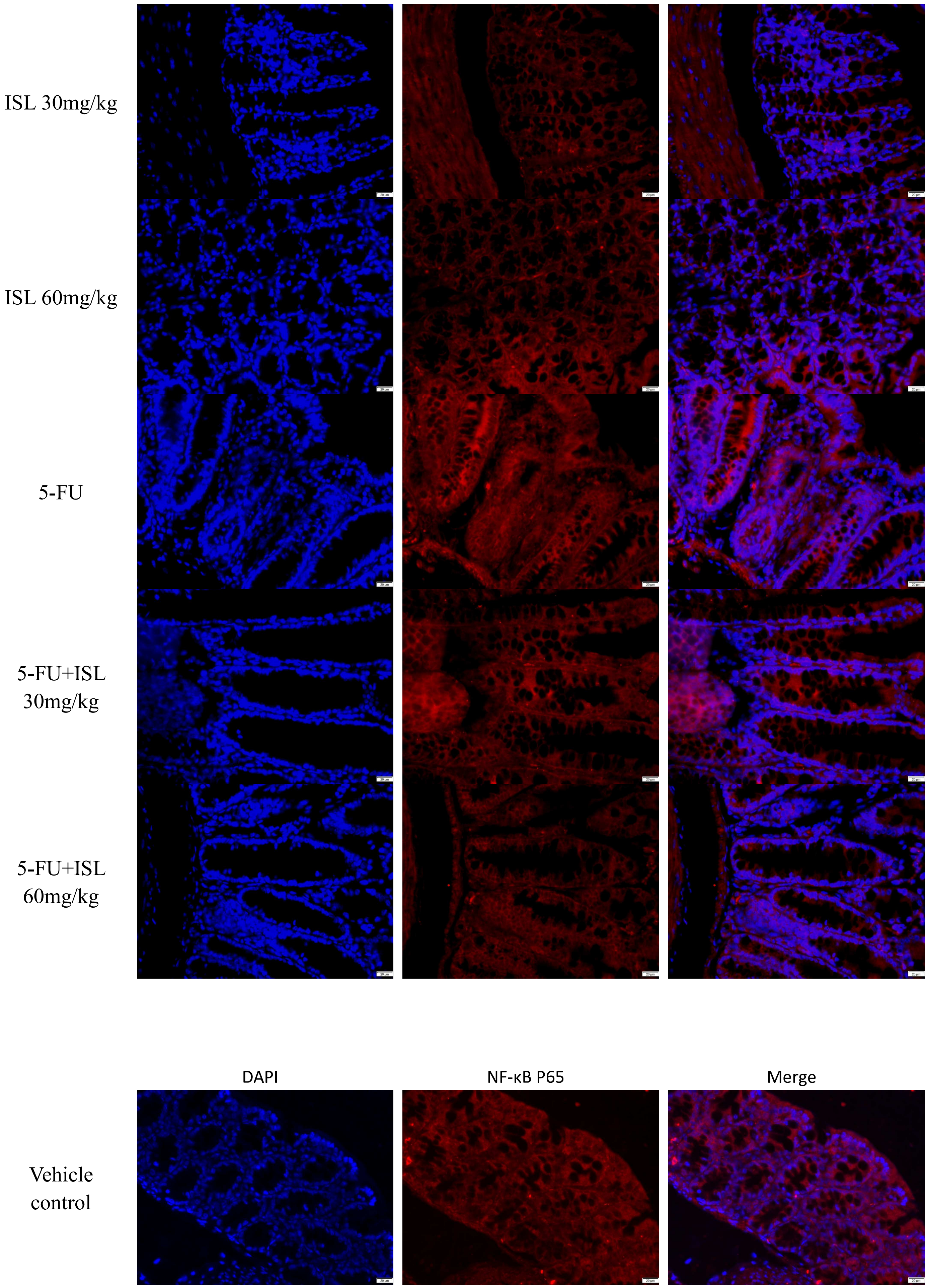
ISL 30mg/kg
ISL 60mg/kg
5-FU
5-FU+ISL 30mg/kg
5-FU+ISL 60mg/kg
DAPI
NF-κB P65
Merge
Vehicle control

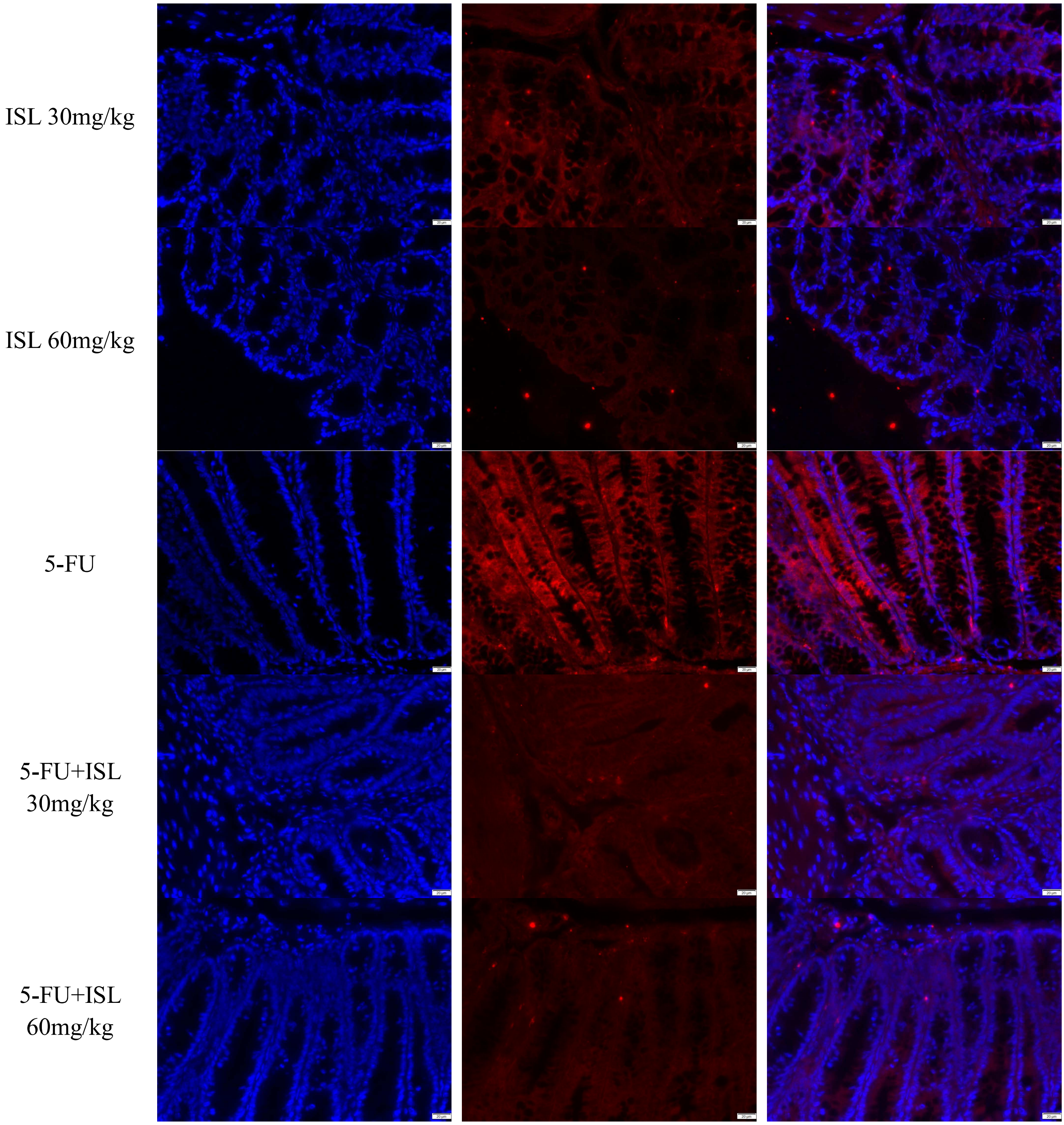
ISL 30mg/kg
ISL 60mg/kg
5-FU
5-FU+ISL 30mg/kg
5-FU+ISL 60mg/kg
