# Supplementary material for: Network analysis and experimental pharmacology study explore the protective effects of Isoliquiritigenin on 5-fluorouracil-Induced intestinal mucositis
Source: Front Pharmacol. 2022 Oct 6;13:1014160. doi: 10.3389/fphar.2022.1014160 (PMC9582754; doi:10.3389/fphar.2022.1014160)

Fig.7A (Results:Effects of ISL on damage of colon and small intestine mucosa induced by 5-FU)

The original images acquired under an fluorescence microscope at 200× magnification as follows.

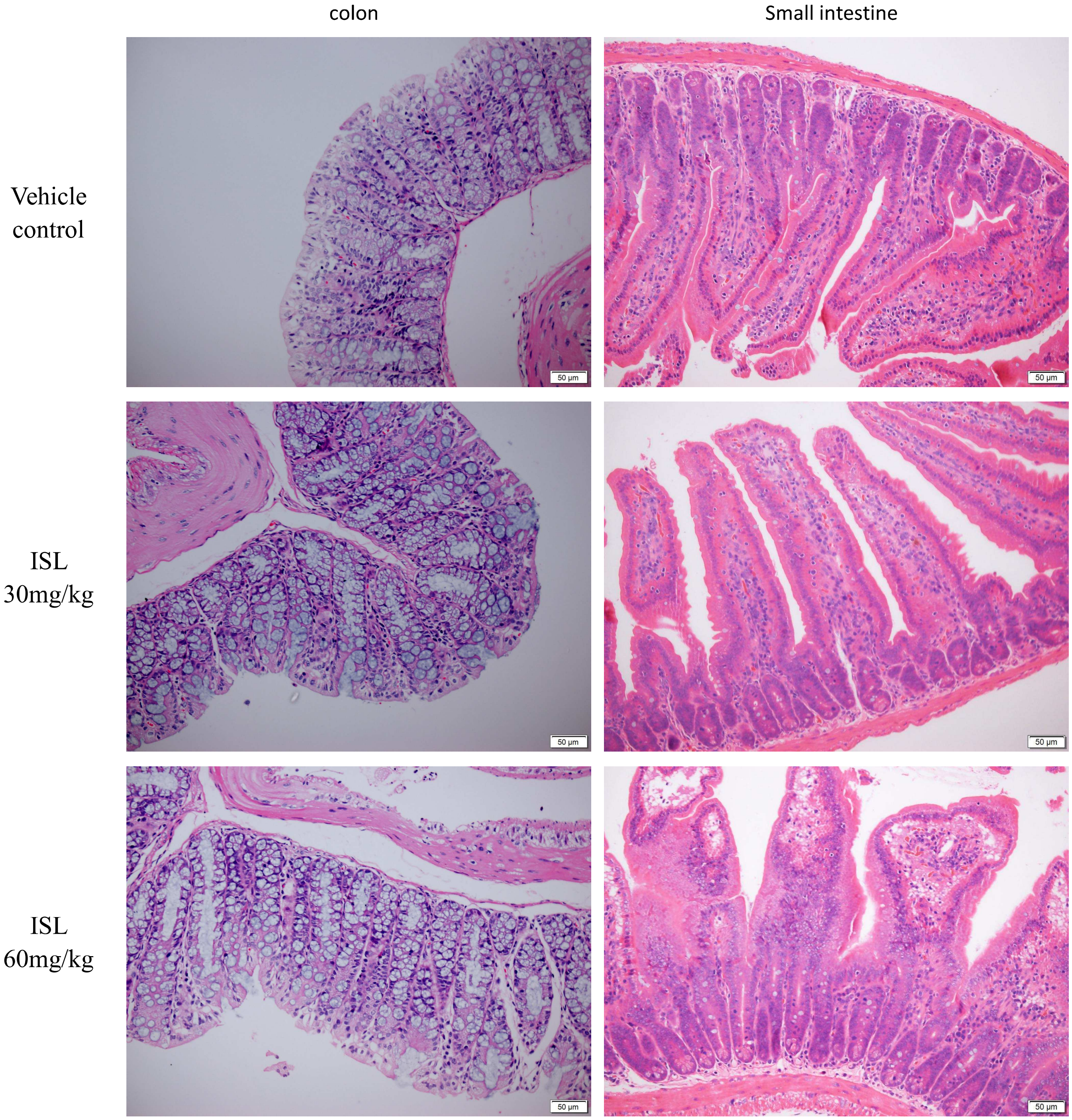

5-FU

5-FU+ISL
30mg/kg

5-FU+ISL
60mg/kg

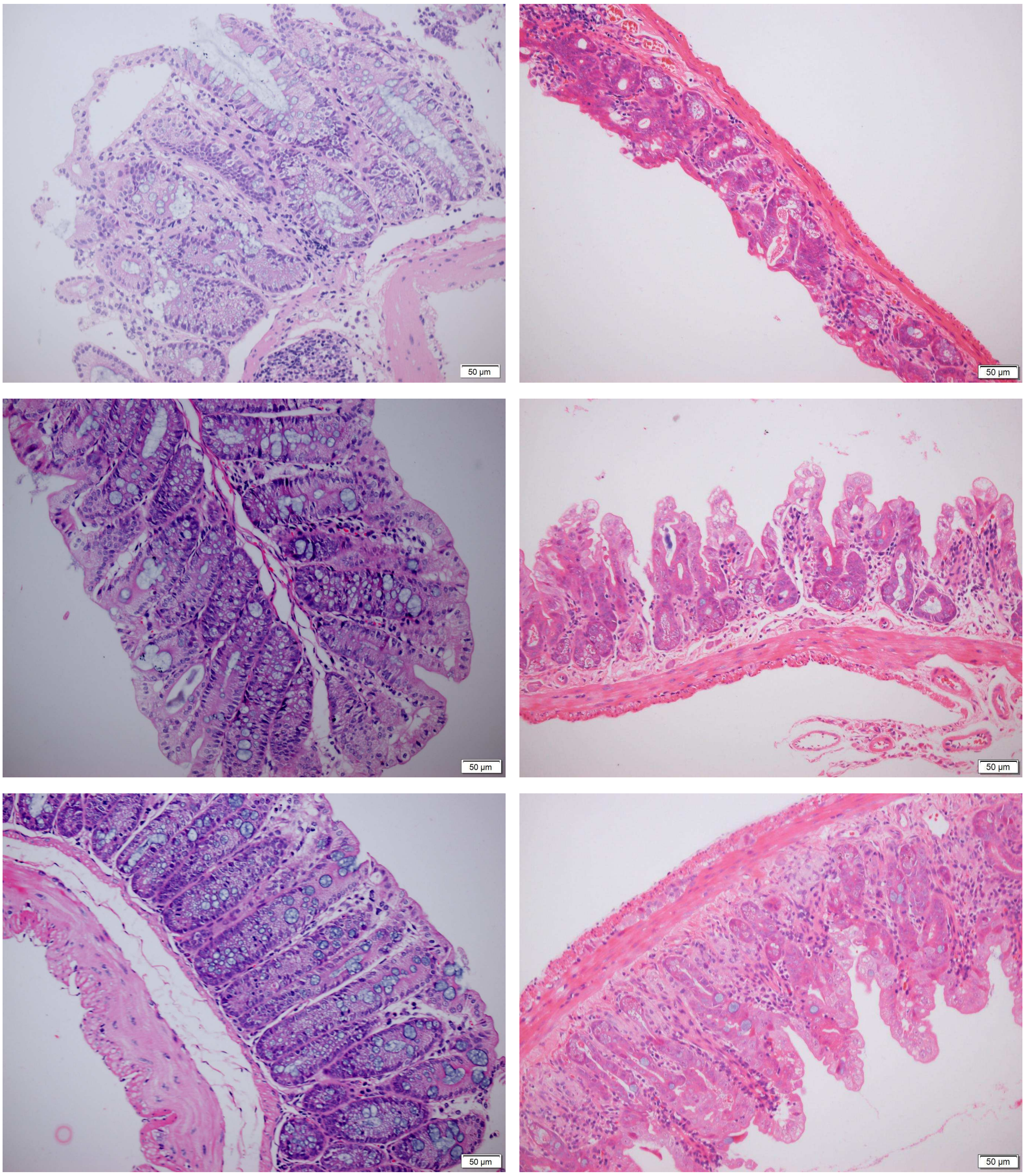

Supplement: Supplementary file 3 [file DataSheet1.PDF]
